# Ginsenoside Rg1 Attenuates Cigarette Smoke-Induced Pulmonary Epithelial-Mesenchymal Transition via Inhibition of the TGF-*β*1/Smad Pathway

**DOI:** 10.1155/2017/7171404

**Published:** 2017-08-13

**Authors:** Sibin Guan, Weiguo Xu, Fengfeng Han, Wen Gu, Lin Song, Wenjing Ye, Qian Liu, Xuejun Guo

**Affiliations:** Department of Respiratory Medicine, Xinhua Hospital, Shanghai Jiao Tong University School of Medicine, 1665 Kongjiang Road, Shanghai 200092, China

## Abstract

Epithelial-mesenchymal transition (EMT) is a process associated with airway remodeling in chronic obstructive pulmonary disease (COPD), which leads to progressive pulmonary destruction.* Panax ginseng* is a traditional herbal medicine that has been shown to improve pulmonary function and exercise capacity in patients with COPD. Ginsenoside Rg1 is one of the main active components and was shown to inhibit oxidative stress and inflammation. The present study investigated the hypothesis that ginsenoside Rg1 attenuates EMT in COPD rats induced by cigarette smoke (CS) and human bronchial epithelial (HBE) cells exposed to cigarette smoke extract (CSE). Our data showed that CS or CSE exposure increased expression of the mesenchymal marker *α*-smooth muscle actin (*α*-SMA) and decreased expression of the epithelial marker epithelial cadherin (E-cad) in both lung tissues and HBE cells, which was markedly suppressed by ginsenoside Rg1. Importantly, CS-induced upregulation of TGF-*β*1/Smad pathway components, including TGF-*β*1, TGF-*β*R1, phospho-Smad2, and phospho-Smad3, was also inhibited by ginsenoside Rg1. Additionally, ginsenoside Rg1 mimicked the effect of SB525334, a TGF-*β*R1-Smad2/3 inhibitor, on suppression of EMT in CSE-induced HBE cells. Collectively, we concluded that ginsenoside Rg1 alleviates CS-induced pulmonary EMT, in both COPD rats and HBE cells, via inhibition of the TGF-*β*1/Smad pathway.

## 1. Introduction

Chronic obstructive pulmonary disease (COPD), characterized by persistent airflow limitation, remains a leading cause of morbidity and mortality worldwide [1]. It is associated with inflammation and fibrosis, leading to airway remodeling and pulmonary dysfunction [2, 3]. Current COPD treatments involving *β*2-agonists, anticholinergics, methylxanthines, corticosteroids, phosphodiesterase-4 inhibitors, rehabilitation, and surgical therapy are effective in reducing symptoms and improving the quality of life of patients with COPD. However, these therapies neither inhibit the progression of COPD towards airway remodeling nor substantively reduce mortality. Therefore, the exploration of novel treatment strategies to prevent airway remodeling is urgently needed.

The mechanisms of airway remodeling in COPD are complex [4]. Recent studies showed that epithelial-mesenchymal transition (EMT), a process in which epithelial cells undergo cytoskeleton reconstruction with loss of cell-cell contacts and acquire a mesenchymal phenotype with excessive extracellular matrix deposition, is involved in the respiratory structural remodeling in COPD [5]. Transitioning epithelial cells acquire enhanced mobility and cross the basal membrane, releasing extracellular matrix proteins that subsequently promote fibrosis and intensify tissue remodeling. Previous studies have demonstrated that the transforming growth factor-*β*1 (TGF-*β*1)/Smad pathway plays a crucial role in triggering EMT [6–8]. The newest study by Mahmood et al. demonstrated that TGF-*β*1 expression was generally higher in COPD subjects throughout the airway wall, while p-Smad2/3 expression was associated with smoking, especially in current smoking COPD; in addition, p-Smad was related to airflow obstruction and S100A4 expression [9]. Phosphorylated Smad2 and Smad3 form a stable complex with Smad4 and then transfer into the nucleus, subsequently modulating the transcription of EMT-associated genes [10]. Besides the Smad cascade, TGF-*β*1 activates EMT via the MEK/ERK, PI3K/AKT, and Wnt/*β*-catenin signaling pathways [11–15]. Accordingly, exploring the mechanism of TGF-*β*1-related pathways in COPD could provide attractive therapeutic targets for inhibiting airway remodeling.

*Panax ginseng*, a traditional herbal medicine, has been widely used in Asian countries for thousands of years. It exhibits multiple pharmacological effects such as antifatigue, anti-inflammation, and antitumor activities, as well as improvement of cognitive function and immunity [16–20]. Interestingly, the compound was also shown to enhance pulmonary function and exercise capacity in patients with COPD [21]. Moreover, Sun et al. found that the bioactive substances of ginseng were distributed to various tissues with the highest level in lung [22]. Ginsenoside Rg1 is one of the main active components of* Panax ginseng* [23], with an oral bioavailability about 18% [24], and has been reported to attenuate cognitive deterioration, oxidative stress, and inflammation [25–27]. In addition, this compound has been shown to inhibit EMT in hepatic carcinoma and renal tubulointerstitial fibrosis via suppression of TGF-*β*1 [28, 29]. However, the role of ginsenoside Rg1 in airway remodeling in COPD remains poorly understood.

In the present study, we investigated the effects and the possible mechanism of ginsenoside Rg1 on cigarette smoke- (CS-) induced pulmonary remodeling, with focus on the TGF-*β*1/Smad pathway. We found that ginsenoside Rg1 suppressed EMT* in vivo* following chronic CS exposure and* in vitro* following cigarette smoke extract (CSE) incubation. These effects were associated with downregulation of TGF-*β*1, TGF-*β*R1, p-Smad2, and p-Smad3. Our findings indicate that ginsenoside Rg1 could act as an attractive therapeutic target for intervention against airway remodeling in COPD.

## 2. Methods

### 2.1. COPD Rat Model and Experimental Protocols

All animal experiments were approved by the Ethics Committee of Xinhua Hospital affiliated to the Shanghai Jiao Tong University School of Medicine (number XHEC-F-2016-014). Rats were raised in the animal center of Xinhua Hospital affiliated to the Shanghai Jiao Tong University School of Medicine. Cigarettes were purchased from the Shanghai Tobacco Company (Da Qian Men cigarettes: 13 mg tar and 1.3 mg nicotine per cigarette, Shanghai, China). Ginsenoside Rg1, with purity exceeding 98%, was obtained from Urchem Sinopharm Chemical Reagent Co. Ltd (Lot number 20150511, Shanghai, China).

Forty, male Sprague-Dawley rats (170–200 g, aged 8–10 weeks, Shanghai SLAC Laboratory Animal Co., Ltd., Shanghai, China) were randomly divided into five groups (*n* = 8 for each group): normal control, COPD group, COPD with low dose of ginsenoside Rg1 (5 mg/kg·d), COPD with medium dose of ginsenoside Rg1 (10 mg/kg·d), and COPD with high dose of ginsenoside Rg1 (20 mg/kg·d). COPD rats (COPD group and Rg1 groups) were placed in 60 L Perspex chambers (4 rats/chamber) and exposed to CS generated from Da Qian Men cigarettes. CS was collected by burning 3 cigarettes at one time, 6 times per day, divided into two 1 h rounds with a 6 h smoke-free interval, 6 days a week, for 12 weeks. Different doses of ginsenoside Rg1 were intragastrically administered 30 min before CS exposure. Normal group and COPD group were intragastrically given normal saline (2 ml per animal). Body weight was measured weekly. All rats were sacrificed at the end of week 12. Blood samples were obtained from the abdominal aorta. The right upper lobe lung was fixed in a 4% neutral formaldehyde solution for pathological and immunohistochemical examinations. The right inferior lobe lung was rapidly reserved in liquid nitrogen and then stored at −80°C for Western blot and real-time PCR analysis.

### 2.2. Cell Culture

#### 2.2.1. CSE Preparation

CSE was prepared by following the method given by Janoff and Carp [30], with modifications. Briefly, a full-length cigarette was combusted through a modified 50 mL syringe apparatus. The smoke was bubbled through 20 mL of serum-free RPMI 1640 medium until the unburned butt was less than 5 mm long. The solution was neutralized with 1 M NaOH to pH 7.4 and then sterilized through a 0.22 *μ*m pore filter. The smoked medium was considered 100% CSE and diluted with RPMI 1640 medium to the desired concentration, which was used within 30 min for each experiment.

#### 2.2.2. Cells Culture and Cell Groups

Human bronchial epithelial cells were grown from a BEAS-2B cell line (number CRL-9609; ATCC, Manassas, VA, USA). HBE cells were cultured in Roswell Park Memorial Institute (RPMI) 1640 medium (HyClone, USA) containing 10% fetal bovine serum (Gibco, Australia) at 37°C with 5% CO_2_ in humidified air. Cell viability was evaluated by a Cell Counting Kit-8 (CCK-8) assay (Dojindo, Kumamoto, Japan). Ginsenoside Rg1 (20 mg) was dissolved in 20 mL RPMI 1640 medium sterilized through a 0.22 *μ*m pore filter and then diluted to the required concentration. Briefly, HBE cells were plated onto 96-well plates at a density of 1 × 10^4^ cells per well and then maintained 24–72 h in 100 *μ*L serum-free medium (SFM) for CCK-8 assay and gene expression detection. When the cells were 60% confluent, they were treated with different concentrations of CSE (0%, 5%, 10%, 15%, and 20%) or ginsenoside Rg1 (5 *μ*M, 10 *μ*M, 20 *μ*M, 40 *μ*M, 80 *μ*M, and 160 *μ*M). After 12, 24, 36, 48, or 72 h, 10 *μ*L of CCK-8 was added to each well, and the plates were returned to the 37°C incubator for 2 h. Absorbance was read at 450 nm using an ELISA reader (ELx800, BioTek Instruments, USA). Pulmonary EMT was induced with either 10% CSE or TGF-*β*1 (10 ng/ml; PeproTech, USA). HBE cells were divided into nine groups: (1) normal, (2) CSE, (3) Rg1 (40 *μ*M), (4) SB525334 (3 *μ*M; Selleck Chem., USA), (5) CSE + Rg1 (40 *μ*M), (6) CSE + SB525334 (3 *μ*M), (7) CSE + Rg1 (40 *μ*M) + SB525334 (3 *μ*M), (8) TGF-*β*1, and (9) TGF-*β*1 + Rg1 (40 *μ*M). After 48 h of incubation, cells were observed using a light microscope, and images were obtained with the Leica Application Suite program. Cells were collected and subjected to Western blot analysis.

### 2.3. Measurement of TGF-*β*1 in Serum

Concentration of TGF-*β*1 in serum was measured by ELISA kits (eBioscience, USA) according to the manufacturer's instructions. The optical density of each sample was read at 45 nm.

### 2.4. Lung Histopathology

The lung tissues were embedded in paraffin and cut into 4 *μ*m thick sections, which were then rehydrated and stained with haematoxylin and eosin to evaluate pulmonary architecture. To access the degree of fibrosis, Masson trichrome staining was performed. Histological changes in each sample were examined under the light microscope. The extent of fibrosis was determined by calculating the blue area normalized to whole pulmonary area.

### 2.5. Real-Time PCR Analysis

Total RNA was extracted from the lung tissue and reverse-transcribed to cDNA following the method as previously described [31]. Primer sequences (synthesized by Sangon Biotech, China) for target genes (E-cad, *α*-SMA, and TGF-*β*1) were as follows: Fwd 5′-TCTCTTGTCCCTTCCACAGC-3′ and Rev 5′-CTCCAGACCCACACCAAAGT-3′ for E-cad; Fwd 5′-TTCGTGACTACTGCTGAGCG-3′ and Rev 5′-CTGTCAGCAATGCCTGGGTA-3′ for *α*-SMA; Fwd 5′-ATTCCTGGCGTTACCTTGG-3′ and Rev 5′-AGCCCTGTATTCCGTCTCCT-3′ for TGF-*β*1. For all lung specimens, glyceraldehyde 3-phosphate dehydrogenase (GAPDH) mRNA (Fwd 5′-GGCACAGTCAAGGCTGAGAATG-3′ and Rev 5′-ATGGTGGTGAAGACGCCAGTA-3′) was amplified as an internal control. To improve the accuracy of real-time PCR results, amplifications were performed in triplicate for each RNA sample.

### 2.6. Western Blot Analysis

Lung tissues and HBE were lysed in RIPA buffer (Beyotime Ins., China) containing 10 *μ*L/mL of phenylmethylsulfonyl fluoride (PMSF) and 10 *μ*L/mL of phosphatase inhibitor. Protein samples were separated via 6–10% sodium dodecyl sulfate polyacrylamide gel electrophoresis (SDS-PAGE) and were transferred to polyvinylidene fluoride membranes (Millipore, Billerica, USA). Membranes were blocked in 5% nonfat milk for 2 h at room temperature and then incubated with primary antibodies against E-cad, *α*-SMA, vimentin, TGF-*β*1, TGF-*β*R1, phospho-Smad2 (p-Smad2), total Smad2, phospho-Smad3 (p-Smad3), or total Smad3 (Abcam, USA) overnight at 4°C. GAPDH (Sigma-Aldrich, USA) was used as the internal control. All primary antibodies could react with both rat and human. After treatment with horseradish peroxidase (HRP) secondary anti-mouse or anti-rabbit antibodies, imaging was performed using enhanced chemiluminescence fluid (Millipore Corporation, USA).

### 2.7. Statistical Analysis

Data were expressed as mean ± standard error of the mean. All graphs and statistical analyses were performed using GraphPad Prism 5 software (GraphPad Software, Inc., La Jolla, CA, USA). One-way ANOVA and Tukey's post hoc test were performed to determine differences among groups. *P* < 0.05 and *P* < 0.01 were considered statistically significant.

## 3. Results

### 3.1. Ginsenoside Rg1 Alleviated Cigarette Smoke-Induced Inflammation and Fibrosis

**Figure 1:**
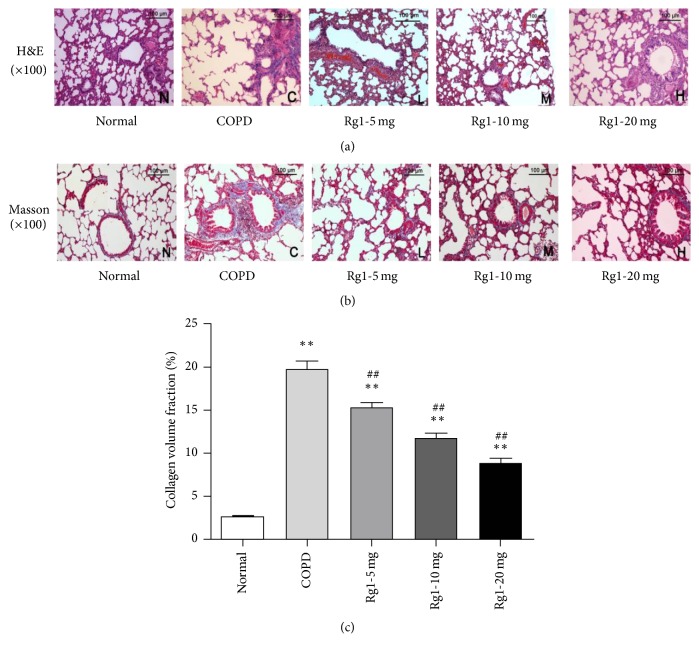
Ginsenoside Rg1 alleviated CS-induced emphysema and fibrosis. (a) Lung histology was analyzed via H&E staining (×100 magnification). (b) Lung histology was analyzed via Masson trichrome staining (×100 magnification). (c) Quantitative analysis of collagen in lung tissue was carried out using Image-Pro Plus 6.0 software. The percentage of the positive staining area of the airway was counted from 6 randomly selected fields per section. Mean optical densities were measured. All data are shown as the mean ± SD; *n* = 6 per group. Statistical significance was assessed by one-way ANOVA and Tukey's post hoc test. ^*∗∗*^*P* < 0.01 versus normal group; ^##^*P* < 0.01 versus COPD group.

COPD is a progressive pulmonary disease that is primarily caused by CS. CS exposure induces chronic inflammation, emphysema, and lung fibrosis, leading to airway remodeling. In comparison to normal group, obvious adhesion, lodging and shedding of cilia, and marked alveolar ectasia were observed in COPD group. These changes were reduced by addition of ginsenoside Rg1 (Figure 1(a)). Masson trichrome staining revealed that, compared with normal group, CS exposure significantly increased pulmonary interstitial fibrosis, which was attenuated with ginsenoside Rg1 treatment (Figure 1(b)). A quantitative analysis produced consistent results (Figure 1(c)). The interstitial fibrosis caused by CS was significantly decreased in ginsenoside Rg1 treated groups (5 mg/kg, 10 mg/kg, and 20 mg/kg) compared with COPD group (15.13%  ± 1.55%, 11.80%  ± 1.39%, and 8.81%  ± 1.69% versus 19.64 ± 2.16%, *P* < 0.01). These data indicated that ginsenoside Rg1 treatment inhibited CS-induced emphysema and airway fibrosis.

### 3.2. Ginsenoside Rg1 Attenuated CS-Induced EMT in Rat Lung Tissue

**Figure 2:**
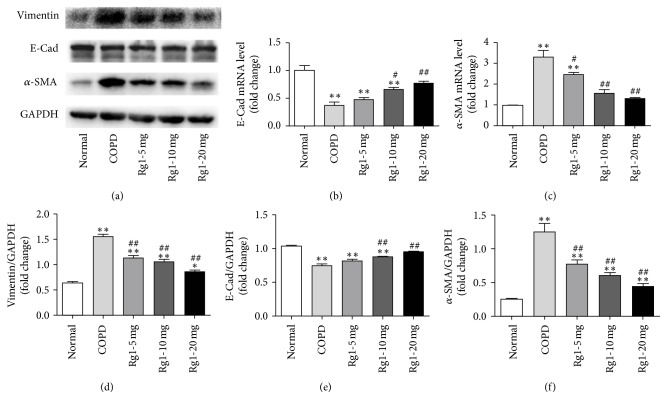
Ginsenoside Rg1 alleviated CS-induced EMT in lung tissues. (a) The expressions of E-cad, *α*-SMA, and vimentin were measured by Western blots ((a), (b), (c), and (d)). The expressions of E-cad and *α*-SMA were measured by real-time PCR ((e) and (f)). All data were shown as the mean ± SD; *n* = 6. Statistical significance was assessed by one-way ANOVA and Tukey's post hoc test. ^*∗*^*P* < 0.05 and ^*∗∗*^*P* < 0.01 versus normal group; ^#^*P* < 0.05 and ^##^*P* < 0.01 versus COPD group.

EMT is characterized by loss of the epithelial marker E-cad and acquisition of the mesenchymal markers *α*-SMA and vimentin. As shown in Figures 2(a)–2(d), Western blots indicated that *α*-SMA and vimentin expressions were markedly increased, whereas E-cad expression decreased slightly in COPD group compared with that in normal group. Ginsenoside Rg1 treatment at 5, 10, and 20 mg/kg markedly decreased *α*-SMA and vimentin expressions and restored E-cad level in a dose-dependent manner (*P* < 0.05 versus COPD group). Real-time PCR showed similar effects of ginsenoside Rg1 on E-cad and *α*-SMA expressions to those observed in Western blots (Figures 2(e) and 2(f)).

### 3.3. Ginsenoside Rg1 Decreased TGF-*β*1 Production in Serum and Suppressed TGF-*β*1/Smad Signaling Expression in Lung Tissue of COPD Rats

**Figure 3:**
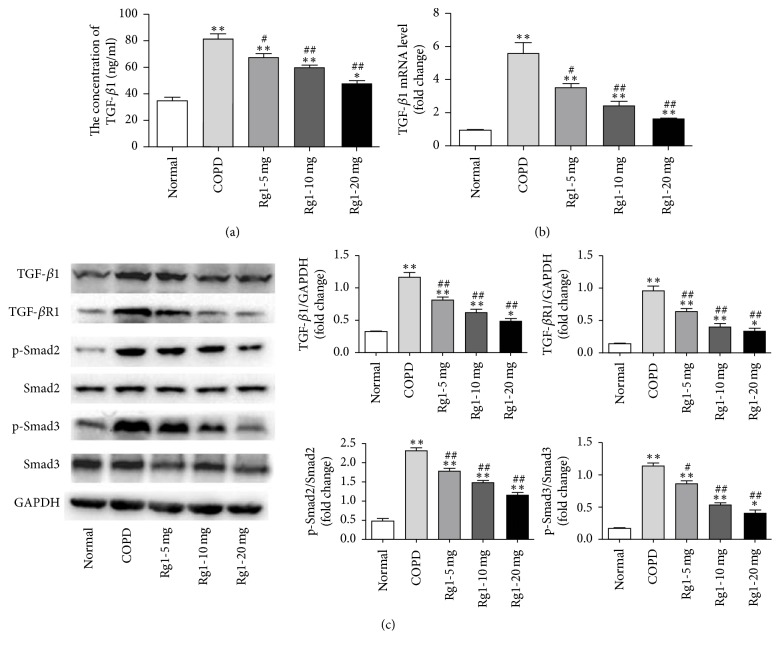
Ginsenoside Rg1 decreased TGF-*β*1 production in serum and suppressed TGF-*β*1/Smad signaling expression in lung tissue of COPD rats. (a) TGF-*β*1 expression in the serum was determined by ELISA. (b) The mRNA level of TGF-*β*1 in lung tissue was determined by real-time PCR. (c) Protein levels of TGF-*β*1, TGF-*β*R1, p-Smad2, total Smad2, p-Smad3, and total Smad3 were determined by Western blot. Data were expressed as the mean ± SD; *n* = 6. Statistical significance was assessed by one-way ANOVA and Tukey's post hoc test. ^*∗*^*P* < 0.05 and ^*∗∗*^*P* < 0.01 versus normal group; ^#^*P* < 0.05 and ^##^*P* < 0.01 versus COPD group.

TGF-*β*—particularly the TGF-*β*1 isoform—plays a key role in driving EMT. Previous studies have confirmed that TGF-*β*1/Smad pathway takes part in EMT. To investigate the potential mechanism by which ginsenoside Rg1 inhibited EMT in the COPD model, we investigated the effects of ginsenoside Rg1 on TGF-*β*1 content in the serum and the mRNA and protein expression of TGF-*β*1 in lung tissue of COPD rats. CS exposure increased serum TGF-*β*1 levels (Figure 3(a)) and enhanced TGF-*β*1 mRNA and protein expression compared with normal group. However, ginsenoside Rg1 at 5, 10, and 20 mg/kg reduced the TGF-*β*1 level in serum (66.98 ± 6.57, 59.53 ± 2.82, and 47.55 ± 4.32 versus 81.03 ± 8.41, *P* < 0.05) and downregulated the mRNA (3.55-, 2.39-, and 1.61-fold versus 5.54-fold, *P* < 0.05) and protein expression of TGF-*β*1 (0.82 ± 0.09, 0.63 ± 0.13, and 0.49 ± 0.07 versus 1.16 ± 0.20, *P* < 0.05) compared with COPD group (Figures 3(b) and 3(c)). We also evaluated different components of TGF-*β*1/Smad signaling in the lung tissues by Western blot. As shown in Figure 3(c), the protein levels of TGF-*β*R1, p-Smad2, and p-Smad3 in COPD group were significantly higher than those of normal group, while ginsenoside Rg1 treatment downregulated expressions of TGF-*β*R1, p-Smad2, and p-Smad3, especially at a high dose. These results suggested that ginsenoside Rg1 attenuated the EMT at least in part through the inhibition of TGF-*β*1 production, which could be associated with the inhibition of TGF-*β*1/Smad signaling pathway.

### 3.4. Cell Viability

**Figure 4:**
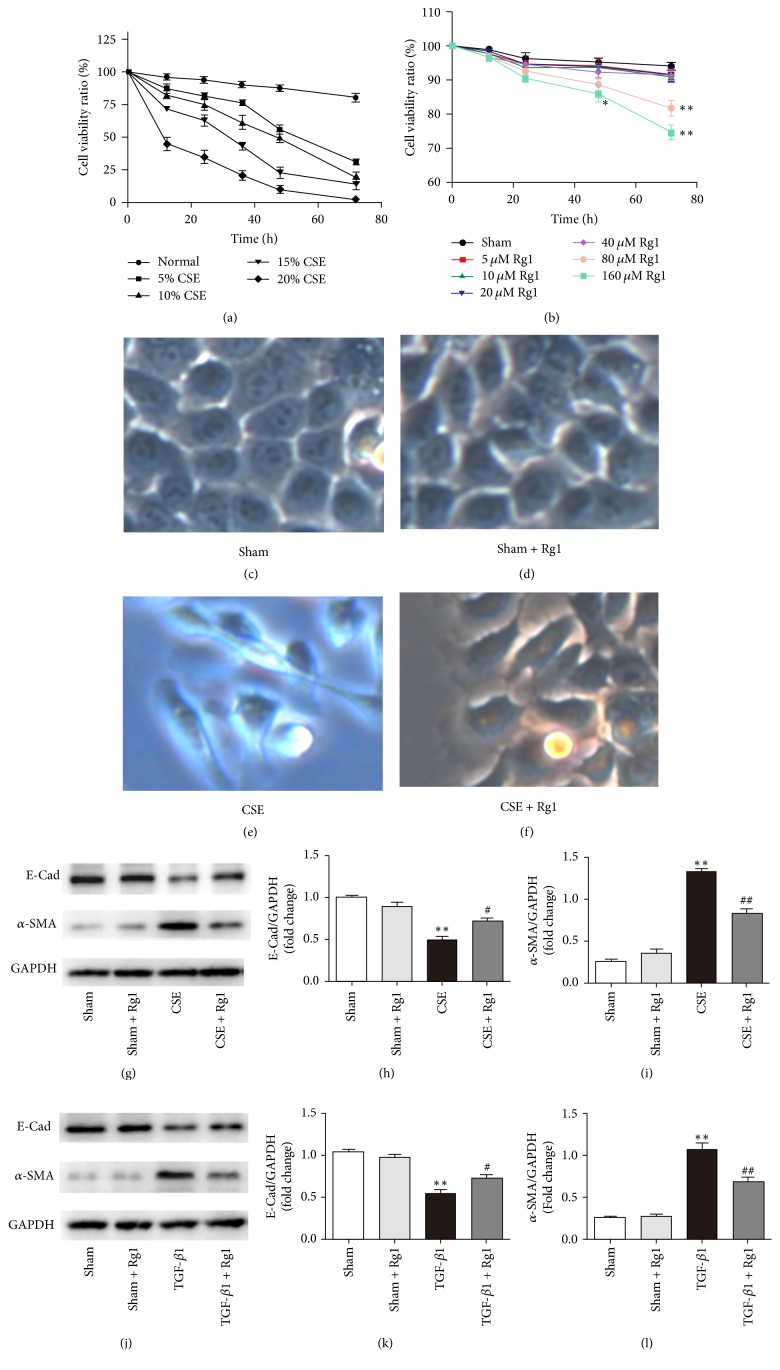
Ginsenoside Rg1 protected HBE cells against CSE or TGF-*β*1-induced EMT. ((a) and (b)) Effects of CSE and ginsenoside Rg1 on the cell viability were measured using a CCK-8 assay. ((c), (d), (e), and (f)) Light microscopy of HBE cells (×100 magnification). ((g), (h), and (i)) HBE cells were induced with 10% CSE and treated with ginsenoside Rg1 (40 *μ*M) for 48 h. Protein expressions of E-cad and *α*-SMA were determined by Western blot. ((j), (k), and (l)) HBE cells were induced with TGF-*β*1 (10 ng/ml) and treated with ginsenoside Rg1 (40 *μ*M) for 48 h. Protein expressions of E-cad and *α*-SMA were determined by Western blot. Data were expressed as the mean ± SD; *n* = 5. Statistical significance was assessed by one-way ANOVA and Tukey's post hoc test. ^*∗*^*P* < 0.05 and ^*∗∗*^*P* < 0.01 versus corresponding untreated controls; ^#^*P* < 0.05 and ^##^*P* < 0.01 versus corresponding ginsenoside Rg1 (−) group.

A CCK-8 assay was used to determine changes in cell viability after HBE cells were incubated with CSE (0%, 5%, 10%, 15%, and 20%) or ginsenoside Rg1 (5 *μ*M, 10 *μ*M, 20 *μ*M, 40 *μ*M, 80 *μ*M, and 160 *μ*M) for 72 h. As shown in Figure 4(a), HBE cells being exposed to CSE exhibited a dose-dependent decrease in cell viability. After 60 h, all CSE groups displayed striking decreases (up to 70%) in the HBE cell viability. CSE incubation greater than 48 h resulted in significant toxicity (>50% viability decline) to HBE cells. Next, we estimated the effect of ginsenoside Rg1 on HBE cell viability. When cells were incubated with 80 *μ*M and 160 *μ*M ginsenoside Rg1 for 72 h, cell viability decreased by 13.2% and 20.7%, respectively (Figure 4(b)). Treatment with 5–40 *μ*M ginsenoside Rg1 showed a minor effect on HBE cell viability. Therefore, HBE cells were exposed to 10% CSE and/or 40 *μ*M ginsenoside Rg1 for 48 h in the following experiments.

### 3.5. Ginsenoside Rg1 Protected HBE Cells against EMT Induced by CSE or TGF-*β*1

To investigate the role of ginsenoside Rg1 in regulating BE cells transdifferentiation, BE cells were stimulated with 10% CSE or TGF-*β*1 (10 ng/mL) in the presence or absence of ginsenoside Rg1 (40 *μ*M) for 48 h. First, we evaluated the morphological changes of HBE cells by light microscopy (40x). The results showed that BE cells changed from cobblestone appearance to long spindle shape in response to CSE exposure. Treatment with ginsenoside Rg1 alleviated the morphological changes (Figures 4(c)–4(f)). Next, HBE cells were collected and subjected to Western blots to detect the protein level of *α*-SMA and E-cad. In accordance with the* in vivo *results, our* in vitro *data indicated that CSE stimulation significantly increased protein expression of *α*-SMA and slightly decreased E-cad protein expression in HBE cells compared with those of the normal group. In the CSE + Rg1 group, the protein level of *α*-SMA was markedly decreased (0.85 ± 0.11 versus 1.34 ± 0.08, *P* < 0.01 versus COPD group), whereas E-cad protein level was increased (0.71 ± 0.09 versus 0.49 ± 0.08, *P* < 0.05 versus COPD group, Figures 4(g)–4(i)). Additionally, compared with TGF-*β*1 alone, ginsenoside Rg1 administration significantly reduced expression of *α*-SMA (0.70 ± 0.07 versus 1.07 ± 0.15, *P* < 0.01 versus COPD group) and upregulated the E-cad level (0.74 ± 0.06 versus 0.55 ± 0.07, *P* < 0.05 versus COPD group). We concluded that ginsenoside Rg1 suppressed the EMT induced by CSE and exhibited similar protective effect on HBE cells treated with TGF-*β*1 (Figures 4(j)–4(l)).

### 3.6. Smad2 and Smad3 Are Activated in CS-Stimulated HBE Cells and Are Inhibited by Ginsenoside Rg1 Treatment

**Figure 5:**
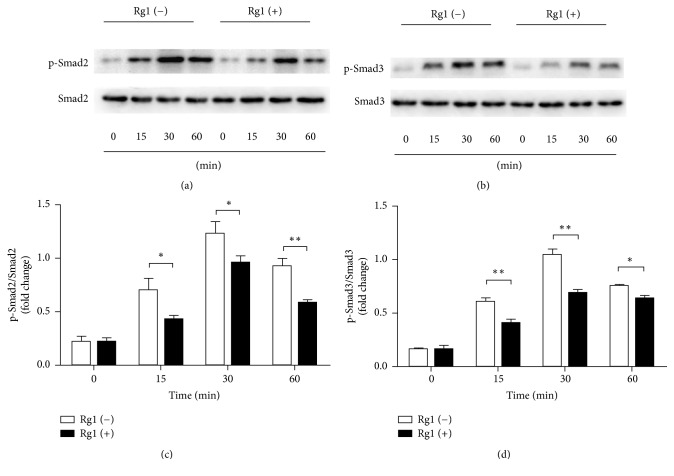
Ginsenoside Rg1 suppressed CS-induced phosphorylation of Smad2 and Smad3 in HBE cells. (a) HBE cells were treated with 10% CSE for 0–60 min; Smad2 was phosphorylated by CSE with maximal phosphorylation at 30 min; the phosphorylation was significantly suppressed by ginsenoside Rg1 (40 *μ*M) treatment. (b) Smad3 was phosphorylated in response to CSE stimulation and peaked at 30 min; the phosphorylation could be abated by ginsenoside Rg1 (40 *μ*M) treatment. Data were expressed as the mean ± SD; *n* = 3 per group. Statistical significance was assessed by two-way ANOVA and Bonferroni's posttest. ^*∗*^*P* < 0.05 and ^*∗∗*^*P* < 0.01.

Previous study demonstrated that* Panax ginseng* could inhibit Smad2 and Smad3 activation [32]. Because the classic signal that regulated EMT is mediated by TGF-*β*1/Smad pathway, we tested whether Smad2 and Smad3 were activated in HBE cells in response to CSE stimulation. HBE cells were treated with 10% CSE for different intervals (0, 15, 30, and 60 min) and then the cell protein was extracted for Western blot analysis of total Smad2, p-Smad2, total Smad3, and p-Smad3. We found that the levels of p-Smad2 and p-Smad3, which were low before CSE stimulation, significantly increased after CSE stimulation and the phosphorylations were the highest at 30 min in response to CSE treatment in HBE cells. By contrast, with ginsenoside Rg1 administration, there was a marked decrease in p-Smad2 and p-Smad3 (Figure 5). Our data suggested that CSE stimulation dramatically enhanced the activation-associated phosphorylation of Smad2 and Smad3. Additionally, ginsenoside Rg1 treatment inhibited the activation of Smad2 and Smad3.

### 3.7. Inhibition of Smad2 and Smad3 Enhanced the Ginsenoside Rg1-Mediated Effect on CS-Induced EMT in HBE Cells

**Figure 6:**
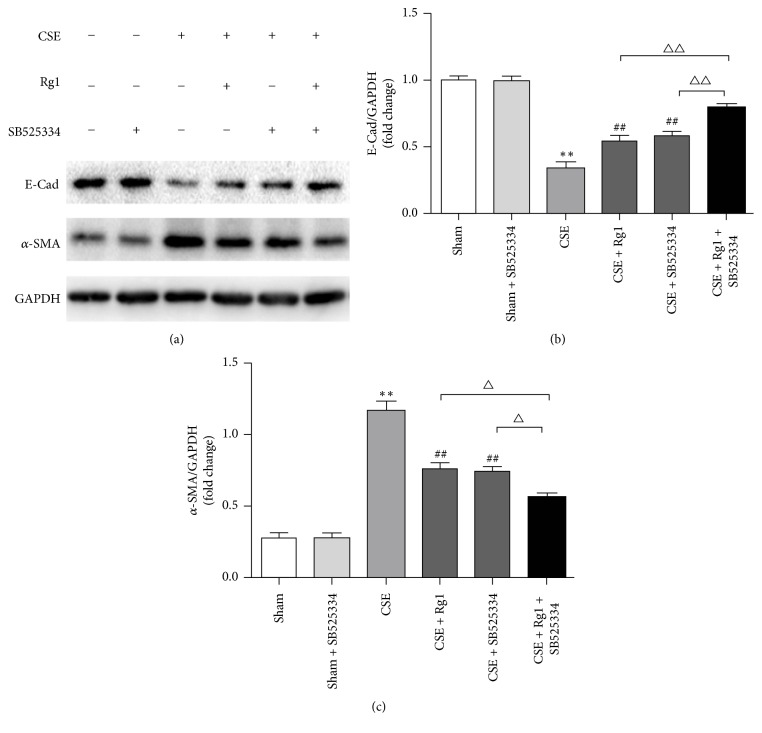
Inhibition of Smad2 and Smad3 abrogated the ginsenoside Rg1-mediated effect on CSE-induced EMT in HBE cells. HBE cells were pretreated with or without SB525334 (3 *μ*M) for 60 min and then stimulated with 10% CSE and cultured with or without ginsenoside Rg1 (40 *μ*M) for 48 h. Protein expressions of E-cad and *α*-SMA were determined by Western blot. Data were expressed as the mean ± SD; *n* = 6. Statistical significance was assessed by one-way ANOVA and Tukey's post hoc test. ^*∗∗*^*P* < 0.01 versus Sham group; ^##^*P* < 0.01 versus CSE group. ^△^*P* < 0.05 and ^△△^*P* < 0.01 versus CSE + Rg1 + SB525334 group.

To further explore the effects of ginsenoside Rg1 on the regulation TGF-*β*1/Smad pathway, we investigated the effect of ginsenoside Rg1 on EMT using SB525334, a specific inhibitor of activin receptor-like kinase 5 (ALK5), which could block the activation of Smad2/3. We first noted no marked difference in E-cad or *α*-SMA protein levels among normal HBE cells and HBE cells treated with ginsenoside Rg1 or SB525334. As expected, pretreatment with SB525334 prior to CSE administration markedly suppressed *α*-SMA and increased E-cad expression at protein level, demonstrating that the TGF-*β*1/Smad pathway is responsible for CSE-induced EMT in HBE cells. There was no statistical difference between CSE + SB525334 group and CSE + Rg1 group. Furthermore, the reduction of *α*-SMA and the restoration of E-cad regulated by ginsenoside Rg1 were enhanced by adding SB525334. These data indicated that ginsenoside Rg1 suppressed pulmonary EMT at least partly through TGF-*β*1/Smad inhibition (Figure 6).

## 4. Discussion

Airway remodeling and emphysema trigger progressive impairment of pulmonary function [33]. Emerging evidence suggests that EMT may lead to the genesis of airway remodeling, indicating a close association between EMT and pulmonary function [4, 5]. Gross et al. and Shergis et al. reported the protective effects of ginseng and ginsenosides on lung function and quality of life in COPD [21, 34]. To better understand the effect of ginsenoside Rg1 on airway remodeling, we treated CS-induced COPD rats with ginsenoside Rg1. The present study indicated that ginsenoside Rg1 decreased *α*-SMA expression, restored the decreased expression of E-cad, a specific epithelial marker in lung tissue, and decreased pulmonary fibrosis in COPD rats. These results indicated the inhibitory effect of ginsenoside Rg1 on EMT. Similar effects of ginsenoside Rg1 were observed in HBE cells treated with CSE or TGF-*β*1. Importantly, our findings revealed that these effects were mediated in part through suppression of TGF-*β*1/Smad signaling pathway, suggesting that ginsenoside Rg1 could be a therapeutic target for airway remodeling in COPD.

EMT is a vital process during wound healing and tissue repair but it can also induce complete tissue fibrosis if the damage or inflammation persists, which has been demonstrated in the liver, kidney, and bowel [35–37]. Recent studies have shown that EMT plays a homeostatic role in the airway response to injury and stress and participates in the pathogenesis of COPD, asthma, and idiopathic pulmonary fibrosis [38–40]. In agreement with the work by Mahmood et al. [38], we observed by immunohistochemical staining that rats exposed to CS for 12 weeks exhibited upregulation of *α*-SMA and downregulation of E-cad in lung tissue; moreover, these findings were validated by quantifying RNA and protein levels of these molecules. The above variations* in vivo* could be countered in a dose-dependent manner by ginsenoside Rg1 administration. At the cellular level, Western blot analysis revealed enhanced *α*-SMA expression and decreased E-cad expression in HBE cells treated with CSE or TGF-*β*1, and these effects could be reversed by ginsenoside Rg1. The histological analysis of the hematoxylin and eosin and Masson trichrome-stained sections of COPD rat lung tissue indicated that ginsenoside Rg1 treatment decreased emphysema and interstitial fibrosis induced by CS. Taken together, these findings demonstrate that ginsenoside Rg1 treatment suppresses both CS-induced pulmonary EMT and TGF-*β*1-induced EMT in HBE cells.

TGF-*β*1 has been implicated as a “master switch” in the induction of EMT.* In vitro* studies demonstrated that exposure to TGF-*β*1 induces different pulmonary epithelial cells to acquire a mesenchymal phenotype [41–43]. Activation of the TGF-*β*1/Smad signaling pathway plays a critical role in the pathogenesis of EMT. Kasai et al. reported that the knockdown of Smad2 with siRNA restored the decreased expression of E-cad in A549 cells treated with TGF-*β*1; the researchers concluded that the mechanism involved in alveolar EMT was probably Smad2-dependent [44]. Yu et al. observed that ginsenoside Rg1 attenuated TGF-*β*1-induced EMT in HepG2 cells and might prevent invasion and migration [28]. Cho et al. reported that Rg1 inhibited TGF-*β*1-induced myofibroblast differentiation in nasal polyp-derived fibroblasts [45]. In addition, it has been indicated that the extract of* Panax ginseng* inhibits TGF-*β*1-mediated fibrosis by suppressing the phosphorylation of Smad2 and Smad3 [32]. Likewise, we showed that ginsenoside Rg1 decreased the level of TGF-*β*1 in serum and lung tissue of COPD rats induced by CS exposure. TGF-*β*1-induced EMT is primarily dependent on Smad pathway initiated by TGF-*β*1 binding to its receptor and activation of the receptor complex. The present study showed that ginsenoside Rg1 dose-dependently decreased the protein expression of TGF-*β*1R, which inhibited the signal transduction of TGF-*β*1, and consequently exerted an inhibitory effect on TGF-*β*1-induced EMT. In addition, our data also showed that ginsenoside Rg1 attenuated the activation of Smad2 and Smad3 in vivo and in vitro. Therefore, we believe that the suppression of EMT by ginsenoside Rg1 was at least partly associated with the reduced production of TGF-*β*1 and might be related to inhibition of the TGF-*β*1/Smad signaling pathway. Interestingly, Mahmood et al. found the significant correlations between Smad2/3 and Smad7 expression with both an EMT activity marker and airflow obstruction [9]. Work on Smad7 is included in our future goals. To assess whether ginsenoside Rg1 attenuated EMT independent of the reduction of TGF-*β*1, we investigated the effects of ginsenoside Rg1 on HBE cells treated with TGF-*β*1. The consistent outcome indicated that ginsenoside Rg1 treatment decreased the protein level of *α*-SMA and alleviated the loss of E-cad in HBE cells compared with TGF-*β*1 alone. Moreover, our* in vitro* study demonstrated that ginsenoside Rg1 mimicked the effect of SB525334, an inhibitor of TGF-*β*1-induced Smad2/3 nuclear translocation, on reduction of *α*-SMA and restoration of E-cad. Furthermore, SB525334 enhanced the suppression of EMT in the presence of ginsenoside Rg1, eliminating the involvement of TGF-*β*1/Smad signaling in ginsenoside Rg1-mediated inhibition of EMT, which may provide novel therapeutic targets for COPD.

However, the mechanism of ginsenoside Rg1-evoked TGF-*β*1 downregulation remains unclear. Current research indicated that ginsenoside Rg1 protected primary cultured cortical neurons from A*β*25-35-induced toxicity in a NF-*κ*B-dependent manner [46]. NF-*κ*B activation could stimulate TGF-*β*1 production* in vitro* [47]. Therefore, NF-*κ*B is probably involved in ginsenoside Rg1-mediated decrease in TGF-*β*1 and the underlying mechanisms need to be further elucidated.

It should be noted that the current study has been implemented only on HBE cells* in vivo* and focused on classically described structure changes and proteins in EMT. Moreover, we could not fully determine the origin of fibroblasts/myofibroblasts accumulated in the surrounding bronchial epithelium in COPD. These could be locally produced or bone marrow-derived or even associated with migration and infiltration of mesenchymal cells in vessels around the bronchus. Indeed, we observed that *α*-SMA expression was significantly increased in pulmonary vessels in COPD (Supplementary Figure S1 in Supplementary Material available online at https://doi.org/10.1155/2017/7171404). We will follow up with a detailed analysis of upstream pathways and EMT-related transcription factors* in vivo* and* in vitro*. Additionally, we will further explore the potential mechanism of ginsenoside Rg1 using a cell culture approach including fibroblast cells and vascular endothelial cells.

In conclusion, the present study demonstrated that ginsenoside Rg1 attenuated the progression of EMT, in both CS-induced COPD rat model and HBE cells exposed to CES, which was at least partly mediated by inhibition of the TGF-*β*1/Smad pathway. The exact mechanisms underlying the beneficial effects of ginsenoside Rg1 warrant further investigation. Nevertheless, our current findings indicate that ginsenoside Rg1 could act as an attractive therapeutic target for intervention against airway remodeling in COPD.

## Supplementary Material

Treatment with 3μm SB525334 showed minor effect on BE cells viability.

## Abbreviations

EMT: Epithelial-mesenchymal transition
COPD: Chronic obstructive pulmonary disease
CS: Cigarette smoke
CSE: Cigarette smoke extract
HBE: Human bronchial epithelial
TGF-*β*1: Transforming growth factor-*β*1
*α*-SMA: *α*-Smooth muscle actin
E-cad: Epithelial cadherin.
