# Supplementary figures and images for: Ginsenoside Rg1 Attenuates Cigarette Smoke-Induced Pulmonary Epithelial-Mesenchymal Transition via Inhibition of the TGF-β1/Smad Pathway
Source: Biomed Res Int. 2017 Aug 13;2017:7171404. doi: 10.1155/2017/7171404 (PMC5572594; doi:10.1155/2017/7171404)

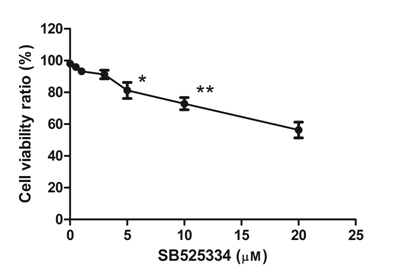

Supplement: Supplementary file 1 — Treatment with 3μm SB525334 showed minor effect on BE cells viability. [file 7171404.f1.docx]
